# Tetra­ethyl­ammonium 2-[bis­(4-hy­droxy­phen­yl)meth­yl]benzoate

**DOI:** 10.1107/S1600536811045016

**Published:** 2011-11-02

**Authors:** Xiaofei Li, Yan Tong, Ching Kheng Quah

**Affiliations:** a College of Pharmacy, Henan University of Traditional Chinese Medicine, Zhengzhou 450008, People’s Republic of China; b X-ray Crystallography Unit, School of Physics, Universiti Sains Malaysia, 11800 USM, Penang, Malaysia

## Abstract

In the title compound, C_8_H_20_N^+^·C_20_H_15_O_4_^−^, the benzoate anions are connected by multiple inter­molecular O—H⋯O hydrogen bonds, forming columns propagating along [1
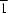
0]. The hydrogen bonding can be described by two rings with *R*_2_^2^(22) and *R*_4_^2^(28) motifs. In the crystal, the tetra­ethyl­ammonium cations are situated between these columns and are linked to them *via* C—H⋯O inter­actions.

## Related literature

Mol­ecules possessing multiple donors or acceptors have long been used to construct different framework structures, which could inter­penetrate and/or include guest mol­ecules, see: Batten *et al.* (2000 ▶); Liu *et al.* (2001 ▶).
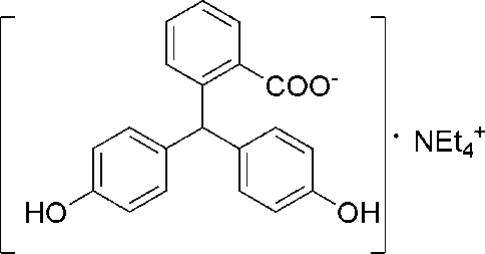


## Experimental

### 

#### Crystal data

C_8_H_20_N^+^·C_20_H_15_O_4_^−^
*M* *_r_* = 449.57
Triclinic, 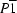

*a* = 9.559 (2) Å
*b* = 10.406 (2) Å
*c* = 14.087 (3) Å
α = 83.390 (3)°
β = 78.711 (3)°
γ = 63.463 (3)°
*V* = 1228.7 (4) Å^3^
*Z* = 2
Mo *K*α radiation
μ = 0.08 mm^−1^
*T* = 298 K
0.28 × 0.16 × 0.16 mm

#### Data collection

Bruker APEXII diffractometer
5826 measured reflections
3869 independent reflections
2847 reflections with *I* > 2σ(*I*)
*R*_int_ = 0.027

#### Refinement

*R*[*F* ^2^ > 2σ(*F* ^2^)] = 0.051
*wR*(*F* ^2^) = 0.136
*S* = 1.04
3869 reflections
298 parameters
H-atom parameters constrained
Δρ_max_ = 0.22 e Å^−3^
Δρ_min_ = −0.22 e Å^−3^

### 

Data collection: *APEX2* (Bruker, 2007 ▶); cell refinement: *SAINT* (Bruker, 2007 ▶); data reduction: *SAINT*; program(s) used to solve structure: *SHELXS97* (Sheldrick, 2008 ▶); program(s) used to refine structure: *SHELXL97* (Sheldrick, 2008 ▶); molecular graphics: *SHELXTL* (Sheldrick, 2008 ▶); software used to prepare material for publication: *publCIF* (Westrip, 2010 ▶).

## Supplementary Material

Crystal structure: contains datablock(s) I, global. DOI: 10.1107/S1600536811045016/su2325sup1.cif

Structure factors: contains datablock(s) I. DOI: 10.1107/S1600536811045016/su2325Isup2.hkl

Supplementary material file. DOI: 10.1107/S1600536811045016/su2325Isup3.cml

Additional supplementary materials:  crystallographic information; 3D view; checkCIF report

## Figures and Tables

**Table 1:** Hydrogen-bond geometry (Å, °)

*D*—H⋯*A*	*D*—H	H⋯*A*	*D*⋯*A*	*D*—H⋯*A*
O1—H1*A*⋯O3^i^	0.82	1.87	2.689 (2)	177
O2—H2*A*⋯O3^ii^	0.82	1.87	2.686 (2)	176
C1—H1*B*⋯O4	0.98	2.23	3.005 (3)	135
C21—H21*A*⋯O2^iii^	0.97	2.56	3.467 (4)	156
C21—H21*B*⋯O4^iv^	0.97	2.39	3.350 (3)	171
C25—H25*B*⋯O4	0.97	2.42	3.382 (3)	172
C27—H27*B*⋯O1^v^	0.97	2.53	3.458 (4)	160

Symmetry codes: (i) 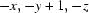; (ii) 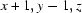; (iii) 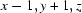; (iv) 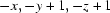; (v) 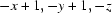.

**Fig. 1.**
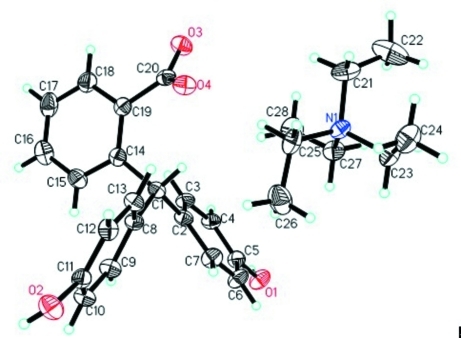
A view of the molecular strcuture of the title compound, showng the numbering scheme and the displacement ellipsoids drawn at the 30% probability level.

**Fig. 2.**
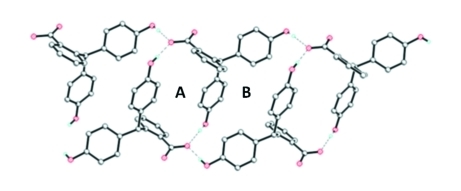
A view of the hydrogen bonded ladder-like column of 2-(bis(4-hydroxyphenyl)methyl)benzoate anions propagating along [110]. The O-H···O hydrogen bonds are shown as dashed lines; C-bound H atoms have been omitted for clarity.

**Fig. 3.**
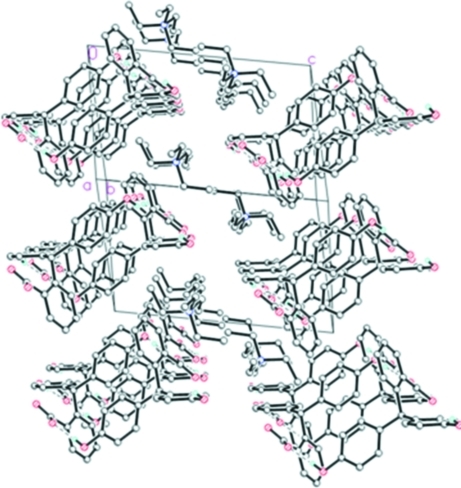
A view of the crystal packing of the title compound. The O-H···O hydrogen bonds are shown as dashed lines; C-bound H atoms have been omitted for clarity.

## supplementary crystallographic information

### Comment

Functional groups containing both hydrogen bond donors and acceptors (OH, CO_2_H) can be used to construct open framework structures, being connected via the various O—H···O hydrogen bonds (Batten *et al.*, 2000; Liu *et al.*, 2001).

In the title compound, the T–shaped 2-(bis(4-hydroxyphenyl)methyl)benzoate anions are connected by four intermolecular O-H···O hydrogen bonds, two hydroxyl groups act as donors, while the carboxyl groups act as acceptor, forming infinite columns propagating along [1 1 0], and two different rectangular grids with ring motifs A [R_2_^2^(22)] and B [R_4_^2^(28)], as shown in Fig. 2.

In the crystal the tetraethylammonium cations are situated between these columns, and are linked to the the carbonyl O atoms of the anions via C-H···O interactions (Table 1).

### Experimental

2-(bis(4-hydroxyphenyl)methyl)benzoic acid (0.25 mmol, 80.0 mg) was dissolved in a water-ethanol (1 ml / 2 ml *v*/*v*) mixture. Tetraethylammonium hydroxide (0.3 mmol, 189.4 mg) was then added. The mixture was set aside for several weeks after which colourless crystals were isolated.

### Refinement

The C-bound H-atoms were included in calculated positions and treated as riding atoms: C-H = 0.93, 0.96, 0.97 and 0.98 Å for CH(aromatic), CH_3_, CH_2_ and CH(methine) H-atoms, respectively, with U_iso_(H) = k × U_eq_(parent C-atom), where k = 1.5 for CH_3_ H-atoms and k = 1.2 for all other H-atoms.

### Crystal data

**Table:**

C_8_H_20_N^+^·C_20_H_15_O_4_^−^	*Z* = 2
*M**_r_* = 449.57	*F*(000) = 484
Triclinic, *P*1	*D*_x_ = 1.215 Mg m^−^^3^
Hall symbol: -P 1	Mo *K*α radiation, λ = 0.71073 Å
*a* = 9.559 (2) Å	Cell parameters from 5859 reflections
*b* = 10.406 (2) Å	θ = 1.5–24.5°
*c* = 14.087 (3) Å	µ = 0.08 mm^−^^1^
α = 83.390 (3)°	*T* = 298 K
β = 78.711 (3)°	Block, colourless
γ = 63.463 (3)°	0.28 × 0.16 × 0.16 mm
*V* = 1228.7 (4) Å^3^	

### Data collection

**Table:**

Bruker APEXII diffractometer	2847 reflections with *I* > 2σ(*I*)
Radiation source: fine-focus sealed tube	*R*_int_ = 0.027
graphite	θ_max_ = 24.5°, θ_min_ = 1.5°
ω scans	*h* = −11→11
5826 measured reflections	*k* = −12→11
3869 independent reflections	*l* = −11→16

### Refinement

**Table:**

Refinement on *F*^2^	Primary atom site location: structure-invariant direct methods
Least-squares matrix: full	Secondary atom site location: difference Fourier map
*R*[*F*^2^ > 2σ(*F*^2^)] = 0.051	Hydrogen site location: inferred from neighbouring sites
*wR*(*F*^2^) = 0.136	H-atom parameters constrained
*S* = 1.04	*w* = 1/[σ^2^(*F*_o_^2^) + (0.0673*P*)^2^ + 0.0071*P*] where *P* = (*F*_o_^2^ + 2*F*_c_^2^)/3
3869 reflections	(Δ/σ)_max_ < 0.001
298 parameters	Δρ_max_ = 0.22 e Å^−^^3^
0 restraints	Δρ_min_ = −0.22 e Å^−^^3^

### Special details

**Table:**

Geometry. Bond distances, angles etc. have been calculated using the rounded fractional coordinates. All su's are estimated from the variances of the (full) variance-covariance matrix. The cell esds are taken into account in the estimation of distances, angles and torsion angles
Refinement. Refinement of *F*^2^ against ALL reflections. The weighted *R*-factor *wR* and goodness of fit *S* are based on *F*^2^, conventional *R*-factors *R* are based on *F*, with *F* set to zero for negative *F*^2^. The threshold expression of *F*^2^ > σ(*F*^2^) is used only for calculating *R*-factors(gt) *etc*. and is not relevant to the choice of reflections for refinement. *R*-factors based on *F*^2^ are statistically about twice as large as those based on *F*, and *R*- factors based on ALL data will be even larger.

### Fractional atomic coordinates and isotropic or equivalent isotropic displacement parameters (Å^2^)

**Table:**

	*x*	*y*	*z*	*U*_iso_*/*U*_eq_	
O1	0.42741 (18)	0.45947 (17)	−0.14441 (11)	0.0574 (6)	
O2	0.67184 (17)	−0.29161 (15)	0.38711 (11)	0.0530 (5)	
O3	−0.24103 (17)	0.47994 (15)	0.27934 (11)	0.0505 (5)	
O4	−0.03207 (17)	0.36798 (16)	0.35336 (11)	0.0489 (5)	
C1	0.2330 (2)	0.1947 (2)	0.19965 (15)	0.0363 (7)	
C2	0.2886 (2)	0.2563 (2)	0.10391 (15)	0.0385 (7)	
C3	0.1892 (2)	0.3242 (2)	0.03587 (15)	0.0414 (7)	
C4	0.2324 (2)	0.3936 (2)	−0.04585 (16)	0.0432 (7)	
C5	0.3781 (2)	0.3957 (2)	−0.06298 (16)	0.0435 (7)	
C6	0.4786 (3)	0.3305 (3)	0.00415 (17)	0.0511 (8)	
C7	0.4327 (3)	0.2632 (2)	0.08658 (17)	0.0490 (8)	
C8	0.3606 (2)	0.0671 (2)	0.24511 (14)	0.0361 (7)	
C9	0.4846 (2)	−0.0451 (2)	0.19308 (16)	0.0434 (7)	
C10	0.5900 (2)	−0.1630 (2)	0.23912 (16)	0.0436 (7)	
C11	0.5743 (2)	−0.1733 (2)	0.33854 (16)	0.0390 (7)	
C12	0.4550 (2)	−0.0610 (2)	0.39170 (16)	0.0404 (7)	
C13	0.3495 (2)	0.0567 (2)	0.34502 (15)	0.0391 (7)	
C14	0.0965 (2)	0.1597 (2)	0.19114 (14)	0.0377 (7)	
C15	0.1287 (3)	0.0446 (2)	0.13518 (16)	0.0484 (8)	
C16	0.0113 (3)	0.0096 (3)	0.12106 (18)	0.0572 (9)	
C17	−0.1424 (3)	0.0908 (3)	0.1623 (2)	0.0630 (10)	
C18	−0.1783 (3)	0.2060 (3)	0.21589 (19)	0.0537 (9)	
C19	−0.0616 (2)	0.2432 (2)	0.23203 (15)	0.0387 (7)	
C20	−0.1123 (2)	0.3721 (2)	0.29301 (15)	0.0381 (7)	
N1	0.17485 (19)	0.65104 (18)	0.35963 (12)	0.0416 (6)	
C21	0.0102 (3)	0.7361 (3)	0.41381 (18)	0.0624 (9)	
C22	−0.0585 (4)	0.8960 (3)	0.3953 (2)	0.1051 (12)	
C23	0.2909 (3)	0.7004 (3)	0.38232 (17)	0.0513 (8)	
C24	0.3070 (3)	0.6899 (3)	0.48754 (18)	0.0679 (11)	
C25	0.2216 (3)	0.4949 (2)	0.39128 (18)	0.0556 (9)	
C26	0.3855 (3)	0.3920 (3)	0.3464 (2)	0.0769 (11)	
C27	0.1787 (3)	0.6721 (3)	0.25098 (15)	0.0500 (8)	
C28	0.0662 (3)	0.6351 (3)	0.21141 (19)	0.0690 (11)	
H1A	0.36870	0.47640	−0.18420	0.0860*	
H1B	0.18880	0.27200	0.24590	0.0440*	
H2A	0.70070	−0.36350	0.35600	0.0790*	
H3A	0.09070	0.32290	0.04550	0.0500*	
H4A	0.16250	0.43930	−0.08970	0.0520*	
H6A	0.57710	0.33180	−0.00590	0.0610*	
H7A	0.50100	0.22120	0.13170	0.0590*	
H9A	0.49710	−0.04080	0.12580	0.0520*	
H10A	0.67250	−0.23640	0.20250	0.0520*	
H12A	0.44540	−0.06420	0.45880	0.0480*	
H13A	0.26870	0.13090	0.38190	0.0470*	
H15A	0.23260	−0.01030	0.10650	0.0580*	
H16A	0.03620	−0.06830	0.08390	0.0690*	
H17A	−0.22220	0.06740	0.15370	0.0760*	
H18A	−0.28330	0.26130	0.24240	0.0650*	
H21A	−0.06030	0.70140	0.39680	0.0750*	
H21B	0.01250	0.71680	0.48260	0.0750*	
H22A	−0.16320	0.94040	0.43190	0.1570*	
H22B	−0.06350	0.91700	0.32750	0.1570*	
H22C	0.00740	0.93270	0.41450	0.1570*	
H23A	0.39440	0.64380	0.34570	0.0620*	
H23B	0.25880	0.79970	0.35990	0.0620*	
H24A	0.38220	0.72430	0.49470	0.1020*	
H24B	0.34310	0.59150	0.51020	0.1020*	
H24C	0.20600	0.74720	0.52470	0.1020*	
H25A	0.21470	0.48670	0.46120	0.0670*	
H25B	0.14560	0.46620	0.37510	0.0670*	
H26A	0.40620	0.29600	0.37030	0.1150*	
H26B	0.46210	0.41780	0.36320	0.1150*	
H26C	0.39280	0.39660	0.27730	0.1150*	
H27A	0.15510	0.77180	0.23370	0.0600*	
H27B	0.28560	0.61380	0.21930	0.0600*	
H28A	0.07730	0.65210	0.14250	0.1040*	
H28B	−0.04060	0.69410	0.24050	0.1040*	
H28C	0.09030	0.53570	0.22600	0.1040*	

### Atomic displacement parameters (Å^2^)

**Table:**

	*U*^11^	*U*^22^	*U*^33^	*U*^12^	*U*^13^	*U*^23^
O1	0.0546 (10)	0.0724 (11)	0.0522 (10)	−0.0367 (9)	−0.0148 (8)	0.0210 (8)
O2	0.0531 (9)	0.0440 (9)	0.0541 (10)	−0.0103 (7)	−0.0238 (8)	0.0070 (7)
O3	0.0453 (9)	0.0406 (8)	0.0523 (10)	−0.0056 (7)	−0.0148 (7)	0.0048 (7)
O4	0.0418 (9)	0.0579 (10)	0.0421 (9)	−0.0154 (7)	−0.0106 (7)	−0.0033 (7)
C1	0.0343 (11)	0.0336 (11)	0.0370 (12)	−0.0107 (9)	−0.0068 (9)	−0.0021 (9)
C2	0.0330 (11)	0.0359 (11)	0.0377 (12)	−0.0066 (9)	−0.0075 (9)	−0.0005 (9)
C3	0.0300 (11)	0.0458 (12)	0.0437 (13)	−0.0125 (10)	−0.0079 (9)	0.0025 (10)
C4	0.0356 (12)	0.0465 (13)	0.0405 (13)	−0.0115 (10)	−0.0101 (10)	0.0043 (10)
C5	0.0424 (13)	0.0421 (12)	0.0430 (13)	−0.0172 (10)	−0.0068 (10)	0.0051 (10)
C6	0.0406 (13)	0.0623 (15)	0.0574 (15)	−0.0287 (12)	−0.0167 (11)	0.0135 (12)
C7	0.0442 (13)	0.0556 (14)	0.0476 (14)	−0.0198 (11)	−0.0198 (11)	0.0102 (11)
C8	0.0330 (11)	0.0371 (11)	0.0350 (12)	−0.0119 (9)	−0.0070 (9)	−0.0006 (9)
C9	0.0434 (12)	0.0471 (13)	0.0335 (12)	−0.0139 (11)	−0.0069 (10)	−0.0019 (10)
C10	0.0363 (12)	0.0415 (12)	0.0440 (14)	−0.0089 (10)	−0.0050 (10)	−0.0041 (10)
C11	0.0340 (11)	0.0385 (12)	0.0471 (14)	−0.0158 (10)	−0.0161 (10)	0.0053 (10)
C12	0.0487 (13)	0.0422 (12)	0.0342 (12)	−0.0216 (10)	−0.0112 (10)	−0.0002 (9)
C13	0.0391 (12)	0.0369 (11)	0.0371 (13)	−0.0126 (10)	−0.0048 (9)	−0.0043 (9)
C14	0.0408 (12)	0.0356 (11)	0.0351 (12)	−0.0147 (9)	−0.0124 (9)	0.0074 (9)
C15	0.0487 (14)	0.0416 (13)	0.0503 (14)	−0.0130 (11)	−0.0142 (11)	−0.0020 (11)
C16	0.0728 (18)	0.0464 (14)	0.0590 (16)	−0.0242 (14)	−0.0330 (14)	0.0044 (12)
C17	0.0617 (17)	0.0517 (15)	0.092 (2)	−0.0315 (14)	−0.0395 (15)	0.0111 (14)
C18	0.0401 (13)	0.0477 (14)	0.0757 (18)	−0.0191 (11)	−0.0198 (12)	0.0072 (13)
C19	0.0393 (12)	0.0364 (11)	0.0390 (12)	−0.0153 (10)	−0.0130 (9)	0.0102 (9)
C20	0.0336 (11)	0.0407 (12)	0.0334 (12)	−0.0138 (10)	−0.0029 (9)	0.0094 (9)
N1	0.0383 (10)	0.0495 (11)	0.0395 (11)	−0.0231 (9)	−0.0074 (8)	0.0076 (8)
C21	0.0437 (14)	0.091 (2)	0.0435 (14)	−0.0243 (13)	−0.0033 (11)	0.0037 (13)
C22	0.094 (2)	0.086 (2)	0.074 (2)	0.0121 (19)	−0.0028 (18)	−0.0091 (18)
C23	0.0548 (14)	0.0603 (15)	0.0504 (15)	−0.0364 (12)	−0.0098 (11)	0.0056 (11)
C24	0.0784 (19)	0.091 (2)	0.0543 (17)	−0.0523 (17)	−0.0235 (14)	0.0101 (14)
C25	0.0663 (16)	0.0576 (15)	0.0610 (16)	−0.0412 (13)	−0.0272 (13)	0.0199 (12)
C26	0.085 (2)	0.0493 (15)	0.095 (2)	−0.0190 (15)	−0.0402 (18)	0.0026 (15)
C27	0.0532 (14)	0.0561 (14)	0.0375 (13)	−0.0234 (12)	−0.0059 (11)	0.0061 (11)
C28	0.088 (2)	0.0799 (19)	0.0538 (16)	−0.0460 (17)	−0.0268 (15)	0.0088 (14)

### Geometric parameters (Å, °)

**Table:**

O1—C5	1.368 (3)	C6—H6A	0.9300
O2—C11	1.372 (3)	C7—H7A	0.9300
O3—C20	1.273 (3)	C9—H9A	0.9300
O4—C20	1.237 (3)	C10—H10A	0.9300
O1—H1A	0.8200	C12—H12A	0.9300
O2—H2A	0.8200	C13—H13A	0.9300
N1—C27	1.517 (3)	C15—H15A	0.9300
N1—C21	1.511 (3)	C16—H16A	0.9300
N1—C23	1.511 (4)	C17—H17A	0.9300
N1—C25	1.517 (3)	C18—H18A	0.9300
C1—C2	1.529 (3)	C21—C22	1.503 (4)
C1—C14	1.532 (3)	C23—C24	1.507 (4)
C1—C8	1.524 (3)	C25—C26	1.507 (4)
C2—C7	1.384 (4)	C27—C28	1.507 (4)
C2—C3	1.386 (3)	C21—H21A	0.9700
C3—C4	1.381 (3)	C21—H21B	0.9700
C4—C5	1.376 (3)	C22—H22A	0.9600
C5—C6	1.380 (4)	C22—H22B	0.9600
C6—C7	1.385 (4)	C22—H22C	0.9600
C8—C13	1.386 (3)	C23—H23A	0.9700
C8—C9	1.388 (3)	C23—H23B	0.9700
C9—C10	1.382 (3)	C24—H24A	0.9600
C10—C11	1.376 (3)	C24—H24B	0.9600
C11—C12	1.380 (3)	C24—H24C	0.9600
C12—C13	1.386 (3)	C25—H25A	0.9700
C14—C15	1.394 (3)	C25—H25B	0.9700
C14—C19	1.404 (3)	C26—H26A	0.9600
C15—C16	1.379 (4)	C26—H26B	0.9600
C16—C17	1.371 (4)	C26—H26C	0.9600
C17—C18	1.365 (4)	C27—H27A	0.9700
C18—C19	1.397 (4)	C27—H27B	0.9700
C19—C20	1.514 (3)	C28—H28A	0.9600
C1—H1B	0.9800	C28—H28B	0.9600
C3—H3A	0.9300	C28—H28C	0.9600
C4—H4A	0.9300		
			
C5—O1—H1A	109.00	C11—C12—H12A	120.00
C11—O2—H2A	109.00	C8—C13—H13A	119.00
C23—N1—C27	105.99 (19)	C12—C13—H13A	119.00
C25—N1—C27	111.37 (18)	C14—C15—H15A	119.00
C21—N1—C27	111.27 (19)	C16—C15—H15A	119.00
C21—N1—C23	111.18 (19)	C17—C16—H16A	120.00
C21—N1—C25	106.47 (19)	C15—C16—H16A	120.00
C23—N1—C25	110.64 (19)	C16—C17—H17A	120.00
C2—C1—C14	111.18 (17)	C18—C17—H17A	120.00
C8—C1—C14	110.30 (16)	C19—C18—H18A	119.00
C2—C1—C8	116.36 (17)	C17—C18—H18A	119.00
C1—C2—C3	121.49 (19)	N1—C21—C22	115.6 (2)
C1—C2—C7	121.35 (19)	N1—C23—C24	115.6 (2)
C3—C2—C7	116.66 (19)	N1—C25—C26	114.6 (2)
C2—C3—C4	121.8 (2)	N1—C27—C28	115.7 (2)
C3—C4—C5	120.6 (2)	N1—C21—H21A	108.00
C4—C5—C6	118.9 (2)	N1—C21—H21B	108.00
O1—C5—C6	118.7 (2)	C22—C21—H21A	108.00
O1—C5—C4	122.4 (2)	C22—C21—H21B	108.00
C5—C6—C7	120.0 (3)	H21A—C21—H21B	107.00
C2—C7—C6	122.1 (2)	C21—C22—H22A	110.00
C1—C8—C13	118.85 (17)	C21—C22—H22B	109.00
C9—C8—C13	116.94 (18)	C21—C22—H22C	109.00
C1—C8—C9	124.13 (18)	H22A—C22—H22B	109.00
C8—C9—C10	121.4 (2)	H22A—C22—H22C	110.00
C9—C10—C11	120.82 (19)	H22B—C22—H22C	109.00
O2—C11—C10	122.58 (18)	N1—C23—H23A	108.00
C10—C11—C12	118.84 (19)	N1—C23—H23B	108.00
O2—C11—C12	118.58 (19)	C24—C23—H23A	108.00
C11—C12—C13	120.0 (2)	C24—C23—H23B	108.00
C8—C13—C12	122.01 (19)	H23A—C23—H23B	107.00
C15—C14—C19	118.1 (2)	C23—C24—H24A	109.00
C1—C14—C19	123.21 (17)	C23—C24—H24B	109.00
C1—C14—C15	118.6 (2)	C23—C24—H24C	109.00
C14—C15—C16	122.0 (2)	H24A—C24—H24B	109.00
C15—C16—C17	119.4 (2)	H24A—C24—H24C	109.00
C16—C17—C18	120.0 (3)	H24B—C24—H24C	110.00
C17—C18—C19	121.8 (3)	N1—C25—H25A	109.00
C14—C19—C18	118.6 (2)	N1—C25—H25B	109.00
C14—C19—C20	123.22 (19)	C26—C25—H25A	109.00
C18—C19—C20	118.2 (2)	C26—C25—H25B	109.00
O4—C20—C19	120.31 (18)	H25A—C25—H25B	107.00
O3—C20—O4	123.56 (18)	C25—C26—H26A	109.00
O3—C20—C19	116.11 (18)	C25—C26—H26B	109.00
C8—C1—H1B	106.00	C25—C26—H26C	110.00
C14—C1—H1B	106.00	H26A—C26—H26B	109.00
C2—C1—H1B	106.00	H26A—C26—H26C	110.00
C4—C3—H3A	119.00	H26B—C26—H26C	109.00
C2—C3—H3A	119.00	N1—C27—H27A	108.00
C3—C4—H4A	120.00	N1—C27—H27B	108.00
C5—C4—H4A	120.00	C28—C27—H27A	108.00
C5—C6—H6A	120.00	C28—C27—H27B	108.00
C7—C6—H6A	120.00	H27A—C27—H27B	107.00
C6—C7—H7A	119.00	C27—C28—H28A	109.00
C2—C7—H7A	119.00	C27—C28—H28B	109.00
C8—C9—H9A	119.00	C27—C28—H28C	109.00
C10—C9—H9A	119.00	H28A—C28—H28B	109.00
C9—C10—H10A	120.00	H28A—C28—H28C	110.00
C11—C10—H10A	120.00	H28B—C28—H28C	110.00
C13—C12—H12A	120.00		
			
C21—N1—C25—C26	−178.7 (2)	C3—C4—C5—O1	178.00 (18)
C23—N1—C21—C22	57.8 (3)	O1—C5—C6—C7	−179.0 (2)
C25—N1—C21—C22	178.4 (2)	C4—C5—C6—C7	0.7 (3)
C27—N1—C21—C22	−60.1 (3)	C5—C6—C7—C2	1.2 (4)
C21—N1—C23—C24	58.7 (3)	C1—C8—C9—C10	−175.2 (2)
C25—N1—C23—C24	−59.4 (3)	C9—C8—C13—C12	−1.1 (3)
C27—N1—C23—C24	179.8 (2)	C13—C8—C9—C10	1.3 (3)
C21—N1—C27—C28	−56.7 (3)	C1—C8—C13—C12	175.57 (19)
C23—N1—C25—C26	−57.8 (3)	C8—C9—C10—C11	0.5 (3)
C27—N1—C25—C26	59.8 (3)	C9—C10—C11—O2	176.8 (2)
C23—N1—C27—C28	−177.7 (2)	C9—C10—C11—C12	−2.6 (3)
C25—N1—C27—C28	61.9 (3)	C10—C11—C12—C13	2.7 (3)
C8—C1—C2—C3	151.70 (19)	O2—C11—C12—C13	−176.68 (19)
C2—C1—C8—C13	143.2 (2)	C11—C12—C13—C8	−0.9 (3)
C14—C1—C8—C9	87.4 (2)	C15—C14—C19—C18	0.9 (3)
C14—C1—C8—C13	−89.0 (2)	C15—C14—C19—C20	−179.14 (19)
C8—C1—C2—C7	−36.7 (3)	C1—C14—C19—C18	176.8 (2)
C14—C1—C2—C3	24.3 (2)	C1—C14—C15—C16	−177.6 (2)
C14—C1—C2—C7	−164.05 (18)	C19—C14—C15—C16	−1.4 (3)
C2—C1—C8—C9	−40.4 (3)	C1—C14—C19—C20	−3.2 (3)
C2—C1—C14—C15	70.1 (2)	C14—C15—C16—C17	0.7 (4)
C2—C1—C14—C19	−105.9 (2)	C15—C16—C17—C18	0.7 (4)
C8—C1—C14—C15	−60.6 (2)	C16—C17—C18—C19	−1.2 (4)
C8—C1—C14—C19	123.5 (2)	C17—C18—C19—C14	0.4 (4)
C1—C2—C7—C6	−173.9 (2)	C17—C18—C19—C20	−179.6 (2)
C3—C2—C7—C6	−1.9 (3)	C18—C19—C20—O3	−39.8 (3)
C7—C2—C3—C4	0.9 (3)	C18—C19—C20—O4	138.7 (2)
C1—C2—C3—C4	172.88 (18)	C14—C19—C20—O4	−41.3 (3)
C2—C3—C4—C5	0.9 (3)	C14—C19—C20—O3	140.2 (2)
C3—C4—C5—C6	−1.7 (3)		

### Hydrogen-bond geometry (Å, °)

**Table:**

*D*—H···*A*	*D*—H	H···*A*	*D*···*A*	*D*—H···*A*
O1—H1A···O3^i^	0.82	1.87	2.689 (2)	177
O2—H2A···O3^ii^	0.82	1.87	2.686 (2)	176
C1—H1B···O4	0.98	2.23	3.005 (3)	135
C21—H21A···O2^iii^	0.97	2.56	3.467 (4)	156
C21—H21B···O4^iv^	0.97	2.39	3.350 (3)	171
C25—H25B···O4	0.97	2.42	3.382 (3)	172
C27—H27B···O1^v^	0.97	2.53	3.458 (4)	160

Symmetry codes: (i) −*x*, −*y*+1, −*z*; (ii) *x*+1, *y*−1, *z*; (iii) *x*−1, *y*+1, *z*; (iv) −*x*, −*y*+1, −*z*+1; (v) −*x*+1, −*y*+1, −*z*.

## References

[bb1] Batten, S. R., Hoskins, B. F., Moubaraki, B., Murray, K. S. & Robson, R. (2000). *Chem. Commun.* pp. 1095–1096.

[bb2] Bruker (2007). *APEX2* and *SAINT* Bruker AXS Inc., Madison, Wisconsin, USA.

[bb3] Liu, R., Mok, K. F. & Valiyaveettil, S. (2001). *New J. Chem.* **25**, 890–892.

[bb4] Sheldrick, G. M. (2008). *Acta Cryst.* A**64**, 112–122. 10.1107/S0108767307043930 18156677

[bb5] Westrip, S. P. (2010). *J. Appl. Cryst.* **43**, 920–925.

